# Multipolar representation of protein structure

**DOI:** 10.1186/1471-2105-7-242

**Published:** 2006-05-04

**Authors:** Apostol Gramada, Philip E Bourne

**Affiliations:** 1 Department of Pharmacology, University of California San Diego, La Jolla, CA 92093, USA; 2 San Diego Supercomputer Center University of California San Diego, La Jolla, CA 92093, USA

## Abstract

**Background:**

That the structure determines the function of proteins is a central paradigm in biology. However, protein functions are more directly related to cooperative effects at the residue and multi-residue scales. As such, current representations based on atomic coordinates can be considered inadequate. Bridging the gap between atomic-level structure and overall protein-level functionality requires parameterizations of the protein structure (and other physicochemical properties) in a quasi-continuous range, from a simple collection of unrelated amino acids coordinates to the highly synergistic organization of the whole protein entity, from a microscopic view in which each atom is completely resolved to a "macroscopic" description such as the one encoded in the three-dimensional protein shape.

**Results:**

Here we propose such a parameterization and study its relationship to the standard Euclidian description based on amino acid representative coordinates. The representation uses multipoles associated with residue C*α *coordinates as shape descriptors. We demonstrate that the multipoles can be used for the quantitative description of the protein shape and for the comparison of protein structures at various levels of detail. Specifically, we construct a (dis)similarity measure in multipolar configuration space, and show how such a function can be used for the comparison of a pair of proteins. We then test the parameterization on a benchmark set of the protein kinase-like superfamily. We prove that, when the biologically relevant portions of the proteins are retained, it can robustly discriminate between the various families in the set in a way not possible through sequence or conventional structural representations alone. We then compare our representation with the Cartesian coordinate description and show that, as expected, the correlation with that representation increases as the level of detail, measured by the highest rank of multipoles used in the representation, approaches the dimensionality of the fold space.

**Conclusion:**

The results described here demonstrate how a granular description of the protein structure can be achieved using multipolar coefficients. The description has the additional advantage of being immediately generalizable for any residue-specific property therefore providing a unitary framework for the study and comparison of the spatial profile of various protein properties.

## Background

The functions of a protein are determined by its three dimensional structure. It is believed that the functional space of all proteins can be spanned by combining a rather small number of structural units, termed folds. The number of different folds is small compared to the total number of proteins, of the order of 1000 for globular, water-soluble proteins [1]. This is in contrast with the exponential complexity of the amino acid-level configuration space of proteins. Therefore, for some purposes, the description of the configuration based on amino acid coordinates is over-detailed. The number of degrees of freedom needed for the description, comparison and classification of the macroscopic, biologically-relevant features of proteins is necessarily much smaller than that associated with the collection of amino acids. Selecting the relevant degrees of freedom and defining methods to compare structures in their reduced space is deemed useful.

The comparison of structures is a central component of many research objectives. For formulations of the problem and a review of various methods of comparison see for example [2]. The problem is particularly important in protein classification. Various classification schemes that have been developed thus far (for example SCOP [3], CATH [4,5], FSSP [6]) approach the objective of selecting the degrees of freedom by using a combination of various empirical and/or qualitative descriptors of protein conformations and parameters derived directly from coordinates of corresponding amino acids (inter-distances for example), recognizing implicitly that, for the purpose of describing its function, the conformation of the proteins is highly unstructured in the microscopic (amino acid) configuration space. Despite this, however, when it comes to quantitatively measure the difference between protein structures, in most cases, the measure of choice remains the root mean square deviation (*rmsd*) between aligned atomic coordinates.

A disadvantage of the *rmsd*-based measures is that it assumes a strict one-to-one correspondence between at least the C alpha (C*α*) atom coordinates of the compared proteins, i.e., it requires an alignment at the amino acid level of detail. Unfortunately, there is no unique formulation for the problem of aligning protein sequences from structure and, as a result, the existing methods produce results which differ in details [7]. Moreover, there exist problems where the shape of a certain region in the molecule is what is analyzed in the context of a biological function. For example, binding sites from different proteins need to be compared for the detection of any similarities [8]. In those cases there is no alignment, or the alignment between amino acids is not relevant and the *rmsd *cannot be defined. Thus far, from a shape perspective, the comparison has included only the molecular surface in that region [8,9]. One limitation (induced by its two-dimensional nature) is that some methods of surface representation impose restrictions on what kind of surfaces can be studied (for instance only star-like surface, i.e. surfaces described by functions on a unit sphere [8]). Another disadvantage is that it may be the case that other properties of the site, which are three-dimensional in nature might be relevant in defining the function. For example, the distribution of charge, hydrophobicity, etc deep beneath the surface of the site. These properties can not be included in a natural way when only the surfaces are compared. In other words, the intrinsic behavior of a protein is a combination of its properties defined through both the internal and external structure of the protein. To more correctly represent a protein warrants the search for alternative parameterizations of protein structure. The approach presented here represents such an alternative.

A fold is characterized by structural features at the multi-residue level. Even though these features are easily recognizable visually in many cases, there is no obvious quantitative way to relate them to the underlying atomic coordinates that exactly describe the structure of the protein. That is, atom coordinates only offer a local description of the structure, while the features defining the fold represent global, shape related properties. Starting with the very rich description given by the coordinates of the atoms that make the protein, one would then need a way to distill from this set of coordinates only the information that is directly associated with these general traits. We do not know of the existence of any systematic approach for the elimination of the redundant information, starting from the initial set of coordinates. Here, we adopt an approach that starts from the other end of the spectrum. Instead of starting from the atom description and discarding non-relevant features, we start from the global level with a very coarse description and refine it by adding descriptors for more and more detailed features.

The need for methods that use global descriptors for the comparison of protein structures has been recognized before. One such method [10,11] has been proposed recently that relies on results from knot theory to extract a number of quantitative features of the path of the protein backbone and compare two such paths in the reduced space of this set of features [12]. Here we present another method that is based on a hierarchical set of descriptors for the distribution of atoms in space. The method is general enough so that it can be refined to describe the protein spatial profile with a geometrical level of detail. It can potentially interpolate between the coarsest description of the structure (knowledge of the number of atoms only) and the most detailed description, equivalent in amount of information to the complete set of atom coordinates. This is achieved by using as coordinates the series of multipolar components associated with a given atom property. To be specific, we will refer here to the multipole tensors associated with the mass of C*α *atoms. Note, however, that as long as the property is distributed uniformly over the set of atoms (in our case the same mass is assigned to each atom), the nature of the property is not relevant. The multipoles themselves represent, up to a constant multiplicative factor, a set of parameters describing exclusively the spatial configuration of the atom set.

Our approach can be combined with other important parameters, for example, mass of the residue instead of the C*α*, charge, hydrophobicity or secondary structure conformation. In those cases, the method will enable the description of the spatial profile of those quantities instead of just the pure geometrical configuration of the molecule. Correspondingly, the method will provide a means to quantitatively compare the proteins according to those properties.

The notion of multipoles originates in physics [13] and is closely related to the representation of functions in terms of spherical harmonics [14]. The use of spherical harmonics in biomolecular research is not new. They have been used for example for the representation and rotation of molecular surface and other properties in an efficient way [15,16], for the purpose of molecular docking [17], for the comparison of binding sites in molecules [8] or for the display of molecular surfaces in molecular visualization [18]. The lower order (or rank) multipoles (up to the quadrupole order) have been used before as a signature for the electrostatic field in the comparison of small molecules for the purpose of drug design [19]. Here, we take the approach that the whole set of multipoles can be interpreted as an alternative set of coordinates for the description of the structure of the molecule. We then show how their tensorial properties can be used for the definition of a distance function in protein configuration space.

The organization of the paper is as follows. In the Results and Discussion section we first define the multipoles and present a qualitative motivation for their use as an alternative parameterization of the shape of the protein. Then we show how their tensorial properties can be used to define a distance function in the conformational space. Since the multipoles are dependent on the location and orientation of the system of axes, the following subsection is used to define a "canonical" reference frame to be used for the purpose of comparison. The concluding subsection is devoted to testing the method. We show that, given the biological relevant portion of the structure, the method successfully discriminates between the families in a test set of proteins from the protein kinase-like superfamily [20]. We then study the correlation between the multipole and Cartesian coordinates representations for the same test set and show that, as expected, the correlation increases with the level of detail of the multipole representation approaching the dimensionality of the fold space. We conclude the paper with a discussion of the advantages of the method and of the various directions in which it can be generalized.

## Results and discussion

### Mutlipolar representation

The notion of multipoles comes from physics where they are used to describe the field generated by the spatial distribution of a scalar quantity such as mass (gravitational field) or charge (electrostatic field) density. The potential of the field created by such a quantity, at a given distance outside the region it occupies in space, can be conveniently expressed in the form of a multipole expansion [13]. Each multipole in the series accounts for the contribution of a certain type of deviation of the density field from a spherically symmetric distribution and, in general, the higher the order of the multipole the smaller the spatial scale of the deviation it describes. In this sense, the multipoles can be viewed as descriptors for the shape of the scalar distribution. The use of multipoles as shape descriptors is also closely related to the more general methods of 3D moments used in the field of object recognition in computer science [21-23]. In the above physical example the multipoles of higher orders typically account for relatively small contributions in the force field compared to lower order multipoles, and they may be neglected for many practical purposes. In a similar way, higher order multipoles representing small scale details of the shape, can be ignored in the process of describing protein structures when only a rough comparison is needed. Before giving a formal definition of multipoles in general, let us start by discussing the few lower order multipoles which are more familiar and more widely encountered in the research literature.

Quantitatively, the multipoles associated with the space distribution of a scalar property (density of mass in our example) form a sequence of tensors over the three dimensional position space. The multipole of rank zero, or the monopole, is just the space integral of the scalar property. When the scalar property is the mass density then the monopole is the total mass of the set of atoms.

The multipole of rank one is proportional to the position of the center of mass. We will use it to set the origin of the coordinate system with respect to which all multipoles are calculated. Therefore, in our calculations, the multipole of rank one, (the dipole as it is commonly known), is always going to be a null vector. For completeness, we should mention that for the multipoles of a distribution of charge this can not always be done. If the total charge is zero there may be a non-zero dipole moment that can not be made to vanish by a translation of coordinates. This is however a technical problem and it has been addressed before [19].

The multipole of rank two, or the quadrupole has nine Cartesian components. For our discrete distribution, the components are given by the following expression:

∑α=1N(3xα,ixα,j−rα2δi,j).     (1)
 MathType@MTEF@5@5@+=feaafiart1ev1aaatCvAUfKttLearuWrP9MDH5MBPbIqV92AaeXatLxBI9gBaebbnrfifHhDYfgasaacH8akY=wiFfYdH8Gipec8Eeeu0xXdbba9frFj0=OqFfea0dXdd9vqai=hGuQ8kuc9pgc9s8qqaq=dirpe0xb9q8qiLsFr0=vr0=vr0dc8meaabaqaciaacaGaaeqabaqabeGadaaakeaadaaeWbqaamaabmaabaGaeG4mamJaemiEaG3aaSbaaSqaaGGaciab=f7aHjabcYcaSiabdMgaPbqabaGccqWG4baEdaWgaaWcbaGae8xSdeMaeiilaWIaemOAaOgabeaakiabgkHiTiabdkhaYnaaDaaaleaacqWFXoqyaeaacqaIYaGmaaGccqWF0oazdaWgaaWcbaGaemyAaKMaeiilaWIaemOAaOgabeaaaOGaayjkaiaawMcaaaWcbaGae8xSdeMaeyypa0JaeGymaedabaGaemOta4eaniabggHiLdGccqGGUaGlcaWLjaGaaCzcamaabmaabaGaeGymaedacaGLOaGaayzkaaaaaa@506C@

Here, *δ*_*i,j *_= 1 for *i *= *j *and 0 otherwise. The sum runs over all *N *C*α *atoms in the structure, *x*_*α*,*i *_is the component *i *of the x→α
 MathType@MTEF@5@5@+=feaafiart1ev1aaatCvAUfKttLearuWrP9MDH5MBPbIqV92AaeXatLxBI9gBaebbnrfifHhDYfgasaacH8akY=wiFfYdH8Gipec8Eeeu0xXdbba9frFj0=OqFfea0dXdd9vqai=hGuQ8kuc9pgc9s8qqaq=dirpe0xb9q8qiLsFr0=vr0=vr0dc8meaabaqaciaacaGaaeqabaqabeGadaaakeaacuWG4baEgaWcamaaBaaaleaaiiGacqWFXoqyaeqaaaaa@3009@ position vector (one of the *x, y, z *Cartesian components) and *r*_*α *_represents the length of the x→α
 MathType@MTEF@5@5@+=feaafiart1ev1aaatCvAUfKttLearuWrP9MDH5MBPbIqV92AaeXatLxBI9gBaebbnrfifHhDYfgasaacH8akY=wiFfYdH8Gipec8Eeeu0xXdbba9frFj0=OqFfea0dXdd9vqai=hGuQ8kuc9pgc9s8qqaq=dirpe0xb9q8qiLsFr0=vr0=vr0dc8meaabaqaciaacaGaaeqabaqabeGadaaakeaacuWG4baEgaWcamaaBaaaleaaiiGacqWFXoqyaeqaaaaa@3009@ position vector. For example, the first diagonal component is ∑(3xα2−rα2)=∑(2xα2−yα2−zα2)
 MathType@MTEF@5@5@+=feaafiart1ev1aaatCvAUfKttLearuWrP9MDH5MBPbIqV92AaeXatLxBI9gBaebbnrfifHhDYfgasaacH8akY=wiFfYdH8Gipec8Eeeu0xXdbba9frFj0=OqFfea0dXdd9vqai=hGuQ8kuc9pgc9s8qqaq=dirpe0xb9q8qiLsFr0=vr0=vr0dc8meaabaqaciaacaGaaeqabaqabeGadaaakeaadaaeabqaamaabmaabaGaeG4mamJaemiEaG3aa0baaSqaaGGaciab=f7aHbqaaiabikdaYaaakiabgkHiTiabdkhaYnaaDaaaleaacqWFXoqyaeaacqaIYaGmaaaakiaawIcacaGLPaaacqGH9aqpdaaeabqaamaabmaabaGaeGOmaiJaemiEaG3aa0baaSqaaiab=f7aHbqaaiabikdaYaaakiabgkHiTiabdMha5naaDaaaleaacqWFXoqyaeaacqaIYaGmaaGccqGHsislcqWG6bGEdaqhaaWcbaGae8xSdegabaGaeGOmaidaaaGccaGLOaGaayzkaaaaleqabeqdcqGHris5aaWcbeqab0GaeyyeIuoaaaa@4ECB@ and one of the non-diagonal terms is ∑*x*_*α*_*y*_*α*_.

For higher order multipoles, enumerating the Cartesian components in closed form is not a simple task. Moreover, there is a large number of symmetry properties obeyed by the Cartesian components and therefore keeping all components of a given multipole would be redundant. Instead, more commonly, the *irreducible spherical components *are defined since they have a compact form when represented in terms of spherical harmonic functions and are independent. This makes them suitable for analytical and numerical calculations. Within the rest of the paper we will use the term multipoles to denote these irreducible components which represent the focus of our attention. We will explicitely name the Cartesian components if needed to distinguish them from the *spherical components*.

For a discrete set of *N *atoms the multipoles of rank *l *are defined as:

qlm=∑i=1NrilYlm*(θi,φi)     (2)
 MathType@MTEF@5@5@+=feaafiart1ev1aaatCvAUfKttLearuWrP9MDH5MBPbIqV92AaeXatLxBI9gBaebbnrfifHhDYfgasaacH8akY=wiFfYdH8Gipec8Eeeu0xXdbba9frFj0=OqFfea0dXdd9vqai=hGuQ8kuc9pgc9s8qqaq=dirpe0xb9q8qiLsFr0=vr0=vr0dc8meaabaqaciaacaGaaeqabaqabeGadaaakeaacqWGXbqCdaWgaaWcbaGaemiBaWMaemyBa0gabeaakiabg2da9maaqahabaGaemOCai3aa0baaSqaaiabdMgaPbqaaiabdYgaSbaakiabdMfaznaaDaaaleaacqWGSbaBcqWGTbqBaeaacqGGQaGkaaGcdaqadaqaaGGaciab=H7aXnaaBaaaleaacqWGPbqAaeqaaOGaeiilaWIae8NXdy2aaSbaaSqaaiabdMgaPbqabaaakiaawIcacaGLPaaaaSqaaiabdMgaPjabg2da9iabigdaXaqaaiabd6eaobqdcqGHris5aOGaaCzcaiaaxMaadaqadaqaaiabikdaYaGaayjkaiaawMcaaaaa@4F06@

where *r*_*i*_, *θ*_*i*_, *φ*_*i *_represent the spherical coordinates of atom *i*, *Y*_*lm *_denotes a spherical harmonic function and the * denotes complex conjugation. For the definition and summary properties of these functions, see for example [24]. Here, since we will only consider the C*α *atoms, we set the mass of each atom to unity to simplify the notation. For a set of arbitrary atoms, each term in the sum would be weighted by the mass of the atom (or another scalar property in a generalized case).

The rank *l *can take any integer value from 0 to ∞ and for each given *l *the number *m *can take values in the range -*l*...*l*. Then, the number of irreducible components, specified by the index *m*, increases linearly with the rank *l *of the multipole as 2*l *+ 1. When all multipoles with rank from 0 to *n *are used, the total number of independent components describing the shape of the protein is (*n *+ 1)^2^. As *n *increases, this number approaches the number of Cartesian components of the position vectors of the C*α *atoms. When the two numbers are equal, the description provided by the set of multipolar components for the structure of the protein is of the same level of detail as the original description offered by the atomic coordinates. We fully recover the amount of information provided by those coordinates. When this happens, from a mathematical standpoint the multipole series is just a coordinate transformation and if not singular, at least in principle, we can transform back and forth from one description to the other.

As a last remark, we will note that the set of multipoles that we use here is only a subset of a larger set which, in its entirety, uniquely describes the potential field surrounding the distribution of charge (for example) [13], for a given set of boundary conditions. The formalism that we present can be extended to include any portion of this complete set of multipoles and this leaves open the possibility for further optimization of a protein comparison process. The reason for retaining this particular subset of multipoles is that they describe the field outside the region occupied by the molecule and therefore they are more likely to correlate with its interaction capabilities and consequently its function.

### Constructing a distance function in the protein conformation space

What makes the set of multipoles defined in Eq. (2) a good set of descriptors for comparison purposes is that they form a series of quantities with remarkable symmetry properties. Specifically, for any given rank *l*, the 2*l *+ 1 components *q*_*lm*_, *m *= *l*, *l *-1, ... -*l *+ 1, -*l *form an irreducible tensorial set of order *l *[25]. This means that under regular rotations in the three-dimensional (3D) physical space, these components are transformed according to a well defined induced rotation matrix (see e.g. [26]), in a way similar to the behavior of a 2*l *+ 1 dimensional vectorial quantity. The immediate benefit is that one can apply the regular operations with vectors to the multipoles of a given rank and one can construct invariant quantities following the known rules from Euclidian vectors. In particular, if we denote by **q**_**1 **_the set of all components {qlm}m=−l,l¯
 MathType@MTEF@5@5@+=feaafiart1ev1aaatCvAUfKttLearuWrP9MDH5MBPbIqV92AaeXatLxBI9gBaebbnrfifHhDYfgasaacH8akY=wiFfYdH8Gipec8Eeeu0xXdbba9frFj0=OqFfea0dXdd9vqai=hGuQ8kuc9pgc9s8qqaq=dirpe0xb9q8qiLsFr0=vr0=vr0dc8meaabaqaciaacaGaaeqabaqabeGadaaakeaadaGadaqaaiabdghaXnaaBaaaleaacqWGSbaBcqWGTbqBaeqaaaGccaGL7bGaayzFaaWaaSbaaSqaaiabd2gaTjabg2da9maanaaabaGaeyOeI0IaemiBaWMaeiilaWIaemiBaWgaaaqabaaaaa@3A77@, we can define the length of the multipole of rank *l *using the scalar product:

‖q1‖=(∑m=−llqlmqlm*)1/2=(∑(−1)mqlmql−m)1/2,     (3)
 MathType@MTEF@5@5@+=feaafiart1ev1aaatCvAUfKttLearuWrP9MDH5MBPbIqV92AaeXatLxBI9gBaebbnrfifHhDYfgasaacH8akY=wiFfYdH8Gipec8Eeeu0xXdbba9frFj0=OqFfea0dXdd9vqai=hGuQ8kuc9pgc9s8qqaq=dirpe0xb9q8qiLsFr0=vr0=vr0dc8meaabaqaciaacaGaaeqabaqabeGadaaakeaadaqbdaqaaGqabiab=fhaXnaaBaaaleaacqWFXaqmaeqaaaGccaGLjWUaayPcSdGaeyypa0JaeiikaGYaaabCaeaacqWGXbqCdaWgaaWcbaGaemiBaWMaemyBa0gabeaakiabdghaXnaaDaaaleaacqWGSbaBcqWGTbqBaeaacqGGQaGkaaGccqGGPaqkdaahaaWcbeqaaiabigdaXiabc+caViabikdaYaaaaeaacqWGTbqBcqGH9aqpcqGHsislcqWGSbaBaeaacqWGSbaBa0GaeyyeIuoakiabg2da9iabcIcaOmaaqaeabaGaeiikaGIaeyOeI0IaeGymaeJaeiykaKYaaWbaaSqabeaacqWGTbqBaaGccqWGXbqCdaWgaaWcbaGaemiBaWMaemyBa0gabeaakiabdghaXnaaBaaaleaacqWGSbaBcqGHsislcqWGTbqBaeqaaOGaeiykaKYaaWbaaSqabeaacqaIXaqmcqGGVaWlcqaIYaGmaaaabeqab0GaeyyeIuoakiabcYcaSiaaxMaacaWLjaWaaeWaaeaacqaIZaWmaiaawIcacaGLPaaaaaa@654F@

where the last part of the equation follows from the definition of the multipoles and well known symmetry properties of the spherical harmonic functions [24]. This norm can then be used to define a distance between two structures inside the subspace defined by the multipolar components (say **q**_**1**_, q′1
 MathType@MTEF@5@5@+=feaafiart1ev1aaatCvAUfKttLearuWrP9MDH5MBPbIqV92AaeXatLxBI9gBaebbnrfifHhDYfgasaacH8akY=wiFfYdH8Gipec8Eeeu0xXdbba9frFj0=OqFfea0dXdd9vqai=hGuQ8kuc9pgc9s8qqaq=dirpe0xb9q8qiLsFr0=vr0=vr0dc8meaabaqaciaacaGaaeqabaqabeGadaaakeaaieqacuWFXbqCgaqbamaaBaaaleaacqWFXaqmaeqaaaaa@2F3C@) of a given rank, provided that either the structures have been previously spatially superimposed, or, a "canonical orientation" of the structures has been set in some consistent manner:

*δ*_*l*_(**q**_**1**_, **q**_**1**_**'**) = ||**q**_**1 **_- **q**_**1**_**'**||.     (4)

There is no a priori prescription for combining distances in subspaces of different ranks *l *to construct a global distance function. Such a prescription needs to be extracted from numerical experimentation with the problem to be modeled and/or from more general principles. The alternative discussed here is the result of our tests of the sensitivity and selectivity in discerning protein structures. Since the dimensionality of multipoles differs with *l*, in order to combine distances from different subspaces to construct a global metric one has to first define quantities with the same dimensionality. The solution adopted in this paper, is to redefine the distance in all ranks so that it has the same dimensionality, say dimensionality of length. Except eventually for a general factor (with the dimension of mass in our example), the dimensionality of the multipoles is a power of length equal to their rank. The general factor can be rendered dimensionless by a proper rescaling. Then, one can obtain a quantity with dimension of length by taking an appropriate root of the Euclidian distance as follows:

*d*_*l*_(**q**_**1**_, **q**_**1**_**'**) = ||**q**_**1 **_- **q**_**1**_**'**||^1/*l*^, *l *> 0.     (5)

Note that, once the multipole components have been calculated, any reference to the original Cartesian coordinates disappears from the representation. As a consequence, unlike the *rmsd *which requires a one-to-one correspondence between the set of atoms in the structures compared, the distance in Eq. (5) is defined for arbitrary structures, without any restriction with respect to their number or sequence of amino-acids. Therefore no alignment is implied. In practice, a normalization with respect to the "size" of the structures involved may still be necessary. For that purpose, each multipole in Eq. (5) can be separately rescaled with a factor inversely dependent on the "size" of the corresponding molecule. Then, instead of Eq. (5), we will use



The notation we use in this formula for the "size" dependent factors (|*q*_0_|, |q′0
 MathType@MTEF@5@5@+=feaafiart1ev1aaatCvAUfKttLearuWrP9MDH5MBPbIqV92AaeXatLxBI9gBaebbnrfifHhDYfgasaacH8akY=wiFfYdH8Gipec8Eeeu0xXdbba9frFj0=OqFfea0dXdd9vqai=hGuQ8kuc9pgc9s8qqaq=dirpe0xb9q8qiLsFr0=vr0=vr0dc8meaabaqaciaacaGaaeqabaqabeGadaaakeaacuWGXbqCgaqbamaaBaaaleaacqaIWaamaeqaaaaa@2F3D@|) is motivated by the fact that the multipole of rank 0 (monopole) is up to a constant numerical factor the "size" of the molecule (the number of atoms for example). When the two structures have the same size, the rescaling of the multipoles in Eq. (6) reduces to a rescaling of the distance (5) by a factor inversely proportional to the common size of the two molecules. This is qualitatively equivalent to the 1/*N *factor in the *rmsd *distance (Eq. (10)). It can be shown that Eq. (6) satisfies the triangle inequality and therefore a global distance function which also satisfies the triangle inequality can be defined by adding the distances (multiplied eventually by a weight factor) for all ranks of the multipoles. Here, we use the following formula:



This function will be used as a dissimilarity measure for proteins in our study. The upper limit in the summation is the maximum rank of the multipoles retained in the representation and determines the dimensionality of the representation and, implicitely, its level of detail.

The interest for reduced representations is manifest in the literature. From a shape perspective, similar to ours, such representations emerge in approaches such as that described in [10,11]. From a different perspective, starting from individual atomic coordinates and using an averaging approach, an alternative method is presented in [27].

### Defining a canonical reference frame

The multipoles behave like vectorial quantities and the numerical values of their components depend on the location and orientation of the reference frame. For the comparison of structures to be meaningful, we need to either minimize the distance in Eq. (7) over all rigid transformations (translations and rotations) of one of the molecules, or to choose a standard for the reference frame with respect to which the multipolar configurations of the molecules are calculated [8]. Since the second approach is much more efficient for large scale computations, we chose to test this second alternative.

The problem of choosing such a standard arises in many research areas where 3D systems are involved [21], and various schemes can be found in the literature, depending of the research field. A common choice is to select a system of axes that is placed at the center of mass and having its three orthogonal directions along the eigenvectors of a suitable, symmetric matrix (principal axes reference frame). While the location at the center of mass is natural, its orientation as described above is ambiguous. This prescription is not appropriate in our case since it does not uniquely define the axes: any combination of permutations and inversions of the versors of a given principal axes frame form also a principal axes frame. Since we are using multipoles of ranks higher than the quadrupole (rank two), which are sensitive to these various orientations, we can not allow an arbitrary choice. We need a prescription that uniquely defines a frame. Our choice is based on the use of lower order vectorial (magnetic) counterparts of the multipoles introduced above. Specifically, we start by defining the following vectors:

e→1=∑i=0N−1(x→i+1−x→i)=(x→N−x→0).     (8)
 MathType@MTEF@5@5@+=feaafiart1ev1aaatCvAUfKttLearuWrP9MDH5MBPbIqV92AaeXatLxBI9gBaebbnrfifHhDYfgasaacH8akY=wiFfYdH8Gipec8Eeeu0xXdbba9frFj0=OqFfea0dXdd9vqai=hGuQ8kuc9pgc9s8qqaq=dirpe0xb9q8qiLsFr0=vr0=vr0dc8meaabaqaciaacaGaaeqabaqabeGadaaakeaacuWGLbqzgaWcamaaBaaaleaacqaIXaqmaeqaaOGaeyypa0ZaaabCaeaadaqadaqaaiqbdIha4zaalaWaaSbaaSqaaiabdMgaPjabgUcaRiabigdaXaqabaGccqGHsislcuWG4baEgaWcamaaBaaaleaacqWGPbqAaeqaaaGccaGLOaGaayzkaaGaeyypa0ZaaeWaaeaacuWG4baEgaWcamaaBaaaleaacqWGobGtaeqaaOGaeyOeI0IafmiEaGNbaSaadaWgaaWcbaGaeGimaadabeaaaOGaayjkaiaawMcaaaWcbaGaemyAaKMaeyypa0JaeGimaadabaGaemOta4KaeyOeI0IaeGymaedaniabggHiLdGccqGGUaGlcaWLjaGaaCzcamaabmaabaGaeGioaGdacaGLOaGaayzkaaaaaa@511A@

e→2=12∑i=0N−1(x→i+1+x→i)×(x→i+1−x→i).     (9)
 MathType@MTEF@5@5@+=feaafiart1ev1aaatCvAUfKttLearuWrP9MDH5MBPbIqV92AaeXatLxBI9gBaebbnrfifHhDYfgasaacH8akY=wiFfYdH8Gipec8Eeeu0xXdbba9frFj0=OqFfea0dXdd9vqai=hGuQ8kuc9pgc9s8qqaq=dirpe0xb9q8qiLsFr0=vr0=vr0dc8meaabaqaciaacaGaaeqabaqabeGadaaakeaacuWGLbqzgaWcamaaBaaaleaacqaIYaGmaeqaaOGaeyypa0ZaaSaaaeaacqaIXaqmaeaacqaIYaGmaaWaaabCaeaadaqadaqaaiqbdIha4zaalaWaaSbaaSqaaiabdMgaPjabgUcaRiabigdaXaqabaGccqGHRaWkcuWG4baEgaWcamaaBaaaleaacqWGPbqAaeqaaaGccaGLOaGaayzkaaGaey41aq7aaeWaaeaacuWG4baEgaWcamaaBaaaleaacqWGPbqAcqGHRaWkcqaIXaqmaeqaaOGaeyOeI0IafmiEaGNbaSaadaWgaaWcbaGaemyAaKgabeaaaOGaayjkaiaawMcaaaWcbaGaemyAaKMaeyypa0JaeGimaadabaGaemOta4KaeyOeI0IaeGymaedaniabggHiLdGccqGGUaGlcaWLjaGaaCzcamaabmaabaGaeGyoaKdacaGLOaGaayzkaaaaaa@568B@

The first vector reduces to the relative position of the last amino acid with respect to the first, while the second one is a more complex quantity that is sensitive to the details of the path of the protein backbone. Except for special cases (for example when the two vectors in Eqs. (8, 9) are not well-defined, or they become parallel), these vectors are independent. Then, they can be orthonormalized and the resulting unit vectors will serve as the first two versors of our *canonical *reference frame. The third one will be their cross product.

The "canonical" reference frame defined by Eqs. (8, 9) is unique by construction. However, other unique definitions can be developed [8]. We are not aware of any rigorous prescription for constructing such a reference frame and therefore our choice remains heuristic.

### Testing the multipole representation

To test our representation of protein structure, we performed a number of calculations with the goal of assessing both its discriminatory power and, where meaningful, its correlation with the Cartesian description.

#### Comparing biologically relevant molecules

As already stated, the use of multipoles opens the possibility of protein shape comparisons without the need for a pre-existing amino acid alignment. However, while technically our representation allows for the comparison of arbitrary collections of atoms, in biological applications, such as protein classification, not any comparison will make sense: we need to restrict the comparison to those portions of the proteins which are relevant to the problem, for example, the functional regions. In principle, the multipoles can be used in identifying corresponding domains in structures, however, as of this moment we do not have fully functional tools to do that. Therefore, as a benchmark for testing the method, we use a manual alignment of the catalytic cores from the protein kinase-like superfamily [20]. The set contains 25 typical protein kinases (TPK) and 6 atypical protein kinases (AK) which phosphorylate non-proteins. As has been shown [20] these diverse structures can be traced to a common ancestor, but today their sequence identity is below 15% in some cases and significant structural changes have taken place, particularly in the C-terminal lobe so that a variety of substrates can be phosphorylated using the same ATP gamma-phosphate transference mechanism. These structures represent an excellent test case for structure recognition since the accurate hand curated alignment provides a valuable benchmark.

**Figure 1:**
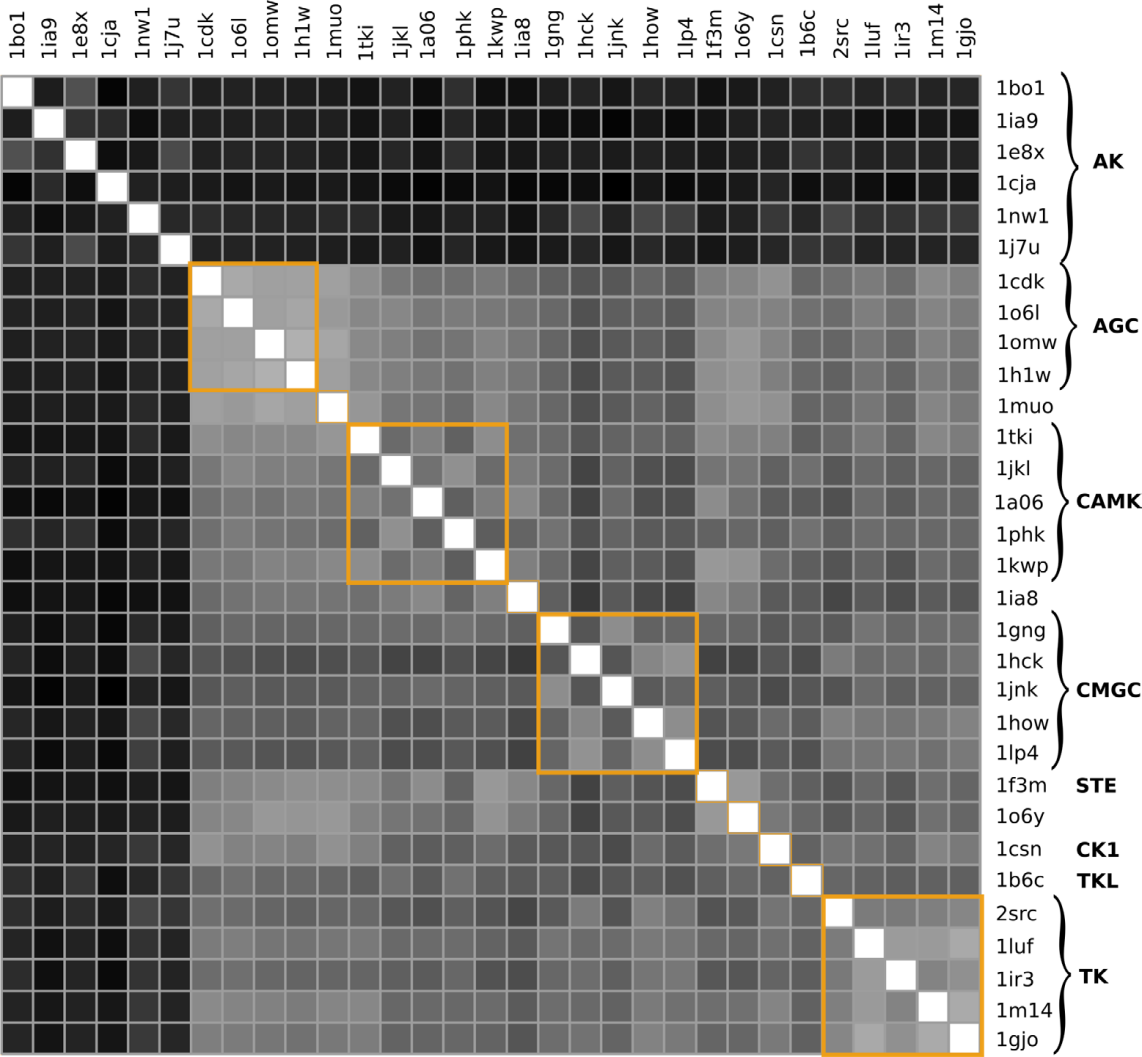
**Matrix of the distances between the biological relevant units of the proteins in the set**. Multipole-based distance matrix calculated from C*α *atoms that are part of the biologically relevant portion of the proteins in the set. The proteins in the set are identified by their pdb id. The groups inside the TPK family are labeled according to the classification produced by Manning et al. [28, 29]. In this calculations all multipoles up to *l*_*max *_= 4 are retained in Eq. (7). Here, and in all other distance matrix representations, darker colors map into bigger distances. The upper six raws and left six columns represent inter-family distances while the rest of the matrix contains only distances between the kinase family members.

We performed an all-against-all comparison of the proteins in the benchmark set, using as input coordinates of C*α *atoms identified as part of the catalytic core [20]. Some of these atoms needed to be omitted because of unresolved portions of some structures as will be discussed later in this section. In this first set of calculations, we did not use any alignment information and there is significant variability in the number of amino acids used for each structure. Eq. (7) was used to generate the distance matrix for all pairs of structures. The results are shown in Figure 1. The ordering of the proteins along the two axis is the one provided by the authors of the set [20]. Therefore, the first six proteins are each a representative of an AK family and the rest are all TPKs. Within the TPKs, the proteins are ordered roughly according to its various groups [28,29] (Figure 1). This implies grouping of all pairs involving an AK kinase in either the upper or left hand band of six rows or columns, respectively. The shading is such that greater distances map into darker squares. A sharp discrimination between any TPK and any AK family on one hand, and between any pair of AK families on the other hand, is observed. Thus, the multipole representation is capable of distinguishing between the major different families included in the test, without the need for detailed alignment and spatial superimposition.

Even at the subfamily level with relatively little shape discrimination, the distance matrix retains some of its discriminatory power. A close examination reveals distinct patterns along the diagonal corresponding to the various groups of kinases in the test set (Figure 1).

The discriminatory power at the family level is limited by unresolved portions of some structures. The lack of coordinates for parts of the polypeptide chain affects the calculated distances both directly (a missing piece of chain is seen as a difference in shape) and indirectly (a missing piece of chain leads to a different canonical reference frame). To reduce these perturbations, we chose to ignore in our calculations any portion of the alignment corresponding to missing parts in at least one of the proteins in the set. Most unresolved portions are relatively short (approximately 20 amino acids) and do not affect the shape dramatically.

#### Correlation with the Cartesian representation

The multipolar description offers a hierarchical approach to characterizing the shape of a molecule. While at the coarsest level there is no information about the shape, except that defined by the length of the chain, at the most refined level of details (when the number of multipole components is of the same order of magnitude as the original Cartesian coordinates) the description is as rich as the original amino acid coordinate set. At this end of the spatial spectrum we would expect a good correlation with results provided by the Cartesian coordinates. To empirically prove this, we need to devise experiments in which both representations can be applied and then compare the results.

An obvious choice is the comparison of aligned proteins, alignment being necessary for the *rmsd *to be defined. Since as yet we do not have tools for aligning proteins based on their multipole representation, we used the high quality expert alignments provided by the authors of the benchmark set [20]. We performed an analysis of how well distances calculated based on the multipole representation compare with the ones based on the Cartesian coordinates. Two different cases were considered, each defined by how the Cartesian and multipolar representation were calculated in the two proteins.

#### Case 1

In the first case, the *rmsd *distances were calculated based on a prior spatial superposition of the aligned structures (the typical approach for assessing structural similarity). The *rmsd*-distance was calculated using the formula

rmsd=1N∑i(x→i−y→i)2.     (10)
 MathType@MTEF@5@5@+=feaafiart1ev1aaatCvAUfKttLearuWrP9MDH5MBPbIqV92AaeXatLxBI9gBaebbnrfifHhDYfgasaacH8akY=wiFfYdH8Gipec8Eeeu0xXdbba9frFj0=OqFfea0dXdd9vqai=hGuQ8kuc9pgc9s8qqaq=dirpe0xb9q8qiLsFr0=vr0=vr0dc8meaabaqaciaacaGaaeqabaqabeGadaaakeaacqWGYbGCcqWGTbqBcqWGZbWCcqWGKbazcqGH9aqpdaGcaaqaamaalaaabaGaeGymaedabaGaemOta4eaamaaqafabaWaaeWaaeaacuWG4baEgaWcamaaBaaaleaacqWGPbqAaeqaaOGaeyOeI0IafmyEaKNbaSaadaWgaaWcbaGaemyAaKgabeaaaOGaayjkaiaawMcaamaaCaaaleqabaGaeGOmaidaaaqaaiabdMgaPbqab0GaeyyeIuoaaSqabaGccqGGUaGlcaWLjaGaaCzcamaabmaabaGaeGymaeJaeGimaadacaGLOaGaayzkaaaaaa@485D@

The two vectors in Eq. (10) denote the coordinates of aligned residues. The multipoles of the aligned portions of the proteins were calculated with the coordinates expressed in the canonical reference frame defined by Eqs. (8, 9).

The multipoles of each protein in a given pair were computed from the C*α *coordinates only. The distances were calculated several times, each time retaining a different range of multipoles to analyze the results at different levels of structural detail. The coarsest calculation corresponds to retaining only multipoles up to rank 2 (quadrupole) and the finest one contains all multipoles up to rank 12. For each set of distances obtained in this way we calculated their linear correlation coefficients with the set of *rmsd *values.

**Figure 2:**
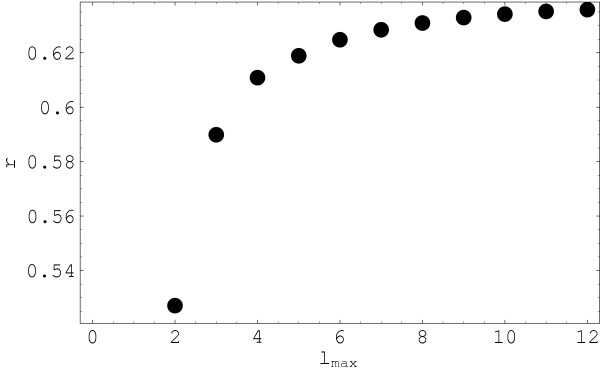
**Correlation coefficient between multipole and rmsd distances**. Correlation coefficient of the multipole and *rmsd*-based distances (r) as a function of the level of detail of the multipole representation (highest multipole rank *l*_*max *_retained in the description). All different pairs of distances from the 31 protein set are included in the comparison. The *rmsd *distances used in this calculation were determined after the spatial superposition of structures. Only aligned residues are included in the calculation of distances.

The correlation calculations are shown in Figure 2 as a function of the highest rank of the multipoles retained in the description, i.e. of the level of detail. In this way, we have an image of how the level of detail of the representation affects the relationship between the two descriptions. The results in Figure 2 are highly significant. The total number of pairs compared in the correlation set is 465 which places even the lowest point on the curve well below a *p *value of 0.001. It is apparent from the correlation curve that, as expected from a theoretical standpoint, as the rank of multipoles retained in the representation increases, the similarity with the *rmsd *results improves. This tendency saturates after a certain level of detail is reached. If the relationship between the two sets of distances was linear, then the saturation would happen at a value equal to unity. In our case, the definition of the multipoles makes the relationship between the two quantities very non-trivial and therefore the linearity between them is, in general, excluded. This is one of the factors that contribute to the saturation of the correlation curve at values smaller than unity.

**Figure 3:**
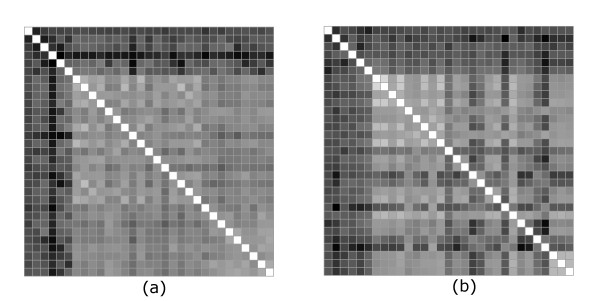
**Comparison between multipole and rmsd distances (*Case 1*)**. The distance matrices between aligned portions of proteins in the multipolar (a) and Cartesian coordinate (b) representations. Multipoles up to order four are retained in (a). The *rmsd *distances are calculated after prior spatial superimposition. The shading is in both cases proportional to the distance, however the scale is normalized to the entire range of values taken in each case. The order of the 31 proteins along the two axis is as described in the text and the same as in Figure 1.

In Figure 3 we show side-by-side the distance matrix in the multipole (a) (where *l*_*max *_= 4, the point of saturation in Figure 2) and Cartesian (b) representations. Again, we see a clear discrimination between the various families in the set, especially in the multipole description. There are a number of extra features inside the TPK family that show up in the Cartesian description and not in our representation. There are also some extra features in the multipole representation, especially inside the AK category, that do not appear in the distance matrix calculated from the Cartesian coordinates. These extra features represent another factor that affects the correlation coefficient. As will be seen from the next calculations, the differences in these details are to a great extent related to the different prescription in the positioning of the proteins, for the purpose of comparison.

**Figure 4:**
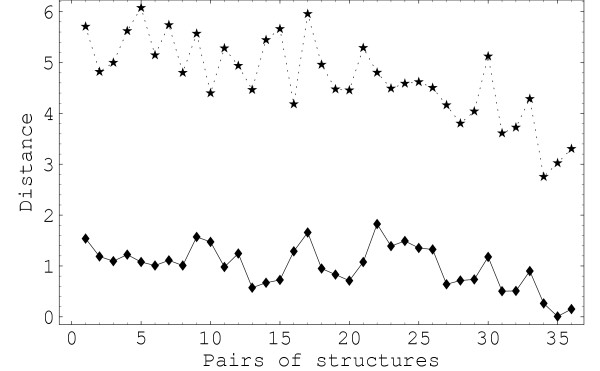
**Distances in a subset of representative proteins**. The distance between all pairs of a subset of representatives: 1bo1, 1ia9, 1e8x, 1cja, 1nw1, 1j7u, 1cdk, 1csn and 1ir3. The upper dashed curve is the *rmsd*. The lowest continuous curve represents the multipoles-based distance rescaled by a factor of 10 with respect to the mean value and shifted to ease comparison. The multiplication factor compensates for the difference in the scale of numerical values of the two measures. In this figure *l*_*max *_= 12.

**Figure 5:**
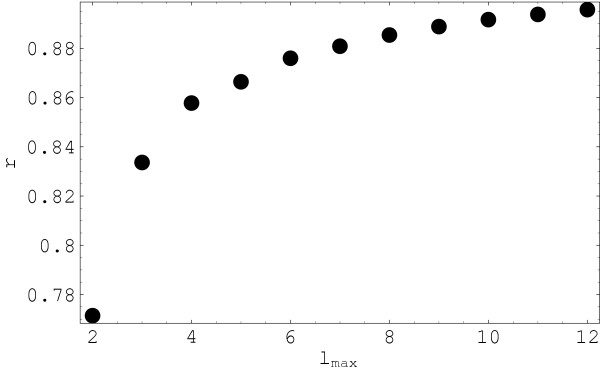
**Correlation coefficient between multipole and rmsd distances for a subset of pairs closer in rmsd**. Correlation coefficient as a function of the level of detail (highest multipole rank *l*_*max *_in the representation). In this calculations only six structures from the smaller set, those occupying the right side in Figure 4 are included: 1cja, 1nw1, 1j7u, 1cdk, 1csn and 1ir3. The distance between pairs of structures in this set are on average smaller than for the whole set in Figure 4.

For a better understanding of how the two descriptions correlate, we need to analyze more carefully the distances in the two representations. To make the discussion more quantitative, in Figure 4 we show the distances between all pairs of non-identical structures in a subset of the original collection of proteins. The subset contains nine structures: 1bol, 1ia9, 1e8x, 1cja, 1nwl, 1j7u, 1cdk, 1csn and 1ir3. The first six are all AK representatives while the last three all belong to the TPK family. The ordering of the 36 resulting independent pairs is as follows: the first structure in the set is paired with all the rest in order; then the second in the set is paired with the rest, etc. For enhanced visibility, we rescaled the multipoles-based distances (after centering at the mean value) and then shifted the whole set of values by the same amount. Both Figure 3 and Figure 4 show that the multipolar distances follow in general big inter-family variations in the *rmsd *distances, but tend to smoothen the intrafamily variations. This is especially obvious inside the TPK family where more pronounced features are visible in the distance matrix in Figure 3b. A closer examinations of Figure 4 also reveals a clearly better correlation in the region of proteins placed closer in our ordering to the TPK family. This region includes roughly all pairs between the subset of structures from 1cja to the end of the list of representatives. The conclusion of a stronger correlation in this area is reinforced if we recalculate the correlation curve with only this subset of structures. The new curve, shown in Figure 5, saturates at values around 0.9 for the correlation coefficient. The major disconnect between the two representations can be attributed to the left hand series of about 15 points in Figure 4. We can see that there is a high variability in the *rmsd *distances between AT and TPK proteins which is not followed accurately by the multipoles-based distance. With our ordering, these are pairs of less related proteins. In this cases we are in the common situation where the *rmsd *in general has little informative value [30] regarding the similarity of the structures and we need to look at global feature in order to characterize similarity. Shape-based descriptors like ours offer a natural path towards a meaningful extension of the notion of similarity.

#### Case 2

**Figure 6:**
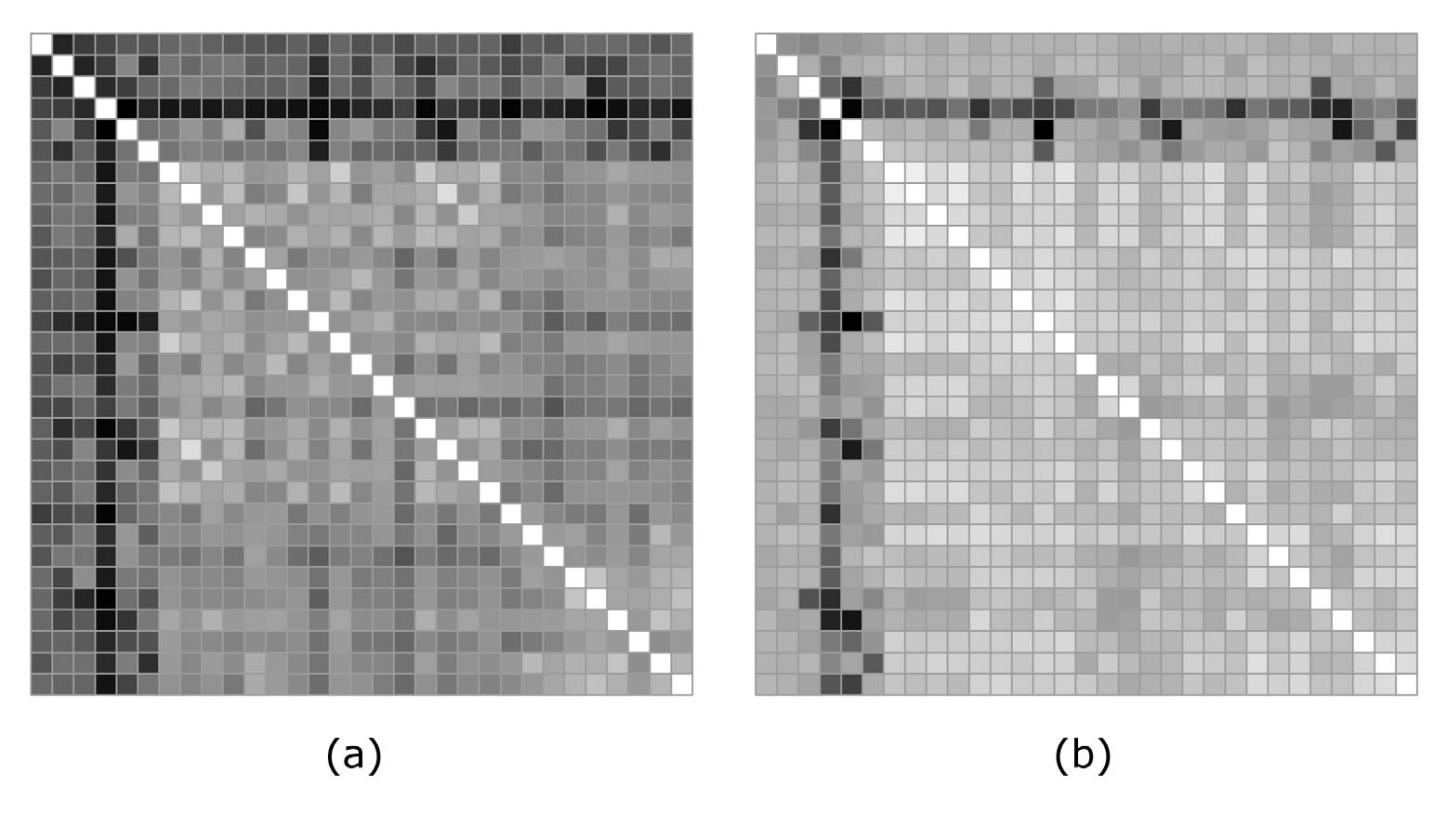
**Comparison between multipole and rmsd distances in the "canonical" frame (*Case 2*)**. Multipole (a) and *rmsd *(b) distance matrices when in both representations proteins in each pair are positioned in the "canonical" reference frame.

**Figure 7:**
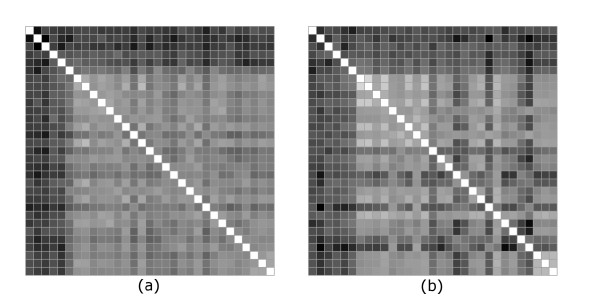
**Comparison between multipole and rmsd distances with superposition (*Case 2*)**. Multipole (a) and *rmsd *(b) distance matrices when in both representations proteins in each pair are superimposed by minimizing the *rmsd *distance.

In a second set of calculations, both the multipole and *rmsd*-based distances were calculated in either the "canonical" reference frame, or after spatial superposition. In the second case the superposition was done by minimizing the *rmsd *between the structures. The results are shown in Figures 6 and 7. There is a visibly higher similarity between the two distance matrices than in Figure 3. This is quantitatively confirmed by a recalculation of the correlation coefficients which show a significant improvement over the previous case.

It is clear that, while the multipole description differs in some intrafamily details from the typical *rmsd *results, especially when the latest are calculated with spatial superposition, the results are quite robust in their capacity to discriminate between the families. The level of correlation increases with the level of detail of the representation, i.e. with the number of multipole approaching the dimensionality of the fold space. These latest results, when compared with the previous ones, suggest that the two ways of positioning the structures for the purpose of comparison are not entirely equivalent and the use of a "canonical" positioning produces a more robust clustering of the structures.

## Conclusion

In this paper we propose a new parameterization of protein structure which provides a new form of characterization and comparison. The approach uses components of the multipoles of consecutive ranks associated with C*α *coordinates.

We have shown:

• Once an approximate "superposition" has been calculated using our canonical reference frame, the multipole distance function is capable of discriminating between protein families.

• The multipole description allows for the adjustment of the level of detail of the comparison and, implicitly, it provides a systematic method for deriving reduced representations of the protein configuration space.

From a biological perspective, our tests show that the comparison based on multipoles is more robust with respect to intrafamily details and the results are more meaningful biologically. From the comparison tests with the Cartesian description, its robustness appears to be related in part to the use of a "canonical" reference frame for the comparison rather than the spatial superposition of the structures. Also, the visible relationship between the distance matrix in Figure 1 and the family classification of the set, in contrast to the distance based on an exact amino acid alignment, suggest that multi-residue shape features are more important to the biological classification than local variations in the alignment. This supports the idea that evolution is more likely driven by shape optimization as required by molecular recognition. Evolutionary events such as sequence insertions and deletions are merely the means to achieve optimal shape complementarity.

For illustration of the multipole method, we used the mass of the C*α *atoms as the relevant physicochemical property. This led to comparison of proteins with respect to their geometrical structure. The method can be refined with minimal changes to use various residue-specific quantities. The only technical difference will be the use of a weight for each term in Eq. (2). The weight is a numerical functions that measure the property of interest, such as hydrophobicity, assigned charge, numerically encoded secondary structure information, etc. It can also represent a composite index reflecting a set of properties assumed to be relevant for investigating a given biological concept.

The use of alternative residue-specific quantities would provide a powerful tool for the comparison of proteins since the residue specific quantities allow an easier discrimination between structures with similar spatial location of the C*α *atoms but differing in local properties of the chain, such as secondary structure conformation for example.

Our plans for further development and extension of the method include:

a) Rigorous definition of the notion of "canonical" reference frame. Our choice, based on features rigidly tied to the set of atoms is inspired by the body reference frames used in physical and engineering-sciences and is intuitive. However, the problem of comparing structures is different and criteria are needed for the identification of "good" reference frames and/or how they affect the protein comparison.

b) Algorithms for fast superposition by minimization of the multipolar distance would be needed as an alternative to the use of a "canonical" reference frame.

c) The definition of a global metric (Eq. 7) contains coefficients controlling the combination of multipoles of various orders. Further optimization of these coefficients for the purpose of protein comparison can lead to biologically more meaningfull metrics.

As a final remark, our representation allows an estimation of the number of degrees of freedom necessary to describe a given class of properties. The saturation of the correlation with the Cartesian representation marks the maximum number of degrees of freedom necessary to "macroscopically" distinguish structures within that class. Since the structure determines the whole biology of the proteins, one can infer from here that the same number of degrees of freedom describes the whole functional space of that class of proteins. The number obtained from such a correlation curve can be used to adjust the dimensionality of the representations used in protein comparisons.

## Methods

The atomic coordinates of the selected members of the protein kinase-like superfamily were obtained from the ASTRAL database [31]. For each pair of proteins, we retain only the C*α *atoms of the biologically relevant parts of the proteins as determined in [20]. The calculations presented in the paper were initially prototyped in Mathematica [32]. A Java program was subsequently written to test the performance of the calculations. We tested the program on a notebook computer with a 1.2 GHz Pentium III processor. The comparison of proteins with about 300 residues, with a level of detail corresponding to *l*_*max *_= 8 takes of the order of 100 ms.

## Authors' contributions

AG developed the formalism, did the calculations and drafted the paper. PB provided the biological context and directed the application and testing of the formalism. Both authors read and approved the final manuscript.
